# The diagnostic challenge of coexisting systemic lupus erythematosus and multiple sclerosis in a young male: Rare but real

**DOI:** 10.1002/ccr3.9521

**Published:** 2024-10-27

**Authors:** Tatiana Charbel, Dany Akiki, Said El Hage, Gaby Moukarzel, Elie Assaf

**Affiliations:** ^1^ Faculty of Medical Sciences Université Saint Joseph Beirut Lebanon; ^2^ Gilbert and Rose‐Marie Chagoury School of Medicine Lebanese American University Beirut Lebanon; ^3^ Resident at the Department of Neurology UF Health Jacksonville Florida USA; ^4^ Department of Internal Medicine Sacred Heart Hospital Beirut Lebanon; ^5^ Department of Neurology Hôtel Dieu de France (HDF) Beirut Lebanon

**Keywords:** autoimmune disease, case study, male, multiple sclerosis, systemic lupus erythematosus

## Abstract

The coexistence of multiple sclerosis (MS) and systemic lupus erythematosus (SLE) is exceptionally rare, especially in males. This case highlights the importance of early diagnosis and treatment with immunomodulatory therapies like rituximab, which can lead to sustained remission in patients with overlapping autoimmune disorders.

## 1 INTRODUCTION

Autoimmune disorders result from a breakdown of immunologic tolerance leading to an immune response against self‐molecules. In most instances the events that initiate the immune response to self‐molecules are unknown, but a number of studies suggest associations with environmental and genetic factors and certain types of infections.^1^, ^2^ Women have a significantly higher risk of developing an autoimmune disease than men, as over 75% of those suffering from autoimmune diseases are female.^2^

Multiple Sclerosis (MS) is a chronic inflammatory disease caused by immune cell infiltration across the blood–brain barrier, responsible for the characteristic multifocal areas of demyelination in the white matter of the brain and the spinal cord. In order to establish a diagnosis, objective evidence of central neurological dysfunction disseminated in space and time (more than one affected area and more than one episode), is required.^3^

Systemic Lupus Erythematosus (SLE), on the other hand, is a B‐cell‐mediated autoimmune disease characterized by the generation of autoantibodies against nuclear antigens and a type III hypersensitivity leading to chronic systemic inflammation and tissue damage of various organs and systems, the central nervous system (CNS) in particular: we call this particular form neuropsychiatric lupus erythematosus (NPSLE).^4^

The presence of both diseases in the same patient is rare, which suggests a relative incompatibility between these diseases. Some cases have already been reported in literature in which the two diseases coexisted. It's noteworthy that, in contrast to our case, every published report of this unusual coexistence involved a female patient.

## 2 CASE HISTORY

A 22‐year‐old male was admitted in August year X with loss of consciousness, severe right leg weakness especially below the knee that exacerbated after the death of a family member. History goes back to two years prior to presentation when he started noticing right sided decreased sensation (especially thermic sensation), in both upper and lower limbs.

His medical history includes a diagnosis of SLE that was made in year X‐3 after an episode of hypertension with bilateral cheek erythema (typical of butterfly rash) and polyarticular inflammatory pain. Initial autoimmune work‐up showed strongly positive Anti‐nuclear antibody (ANA) (1/1280) and negative Anti‐double stranded DNA (Anti‐dsDNA). In addition, Doppler echocardiography revealed cardiac hypertrophy and coarctation of the aorta. Consequently, the patient was discharged on Hydroxychloroquine 200 mg orally three times daily for SLE, Irbesartan 300 mg once daily for hypertension, as well as a statin. While taking a detailed history, the patient was noted to have lactose intolerance in his childhood, and asthma treated with inhalers. He also had a family history of rheumatoid arthritis.

**FIGURE 1:**
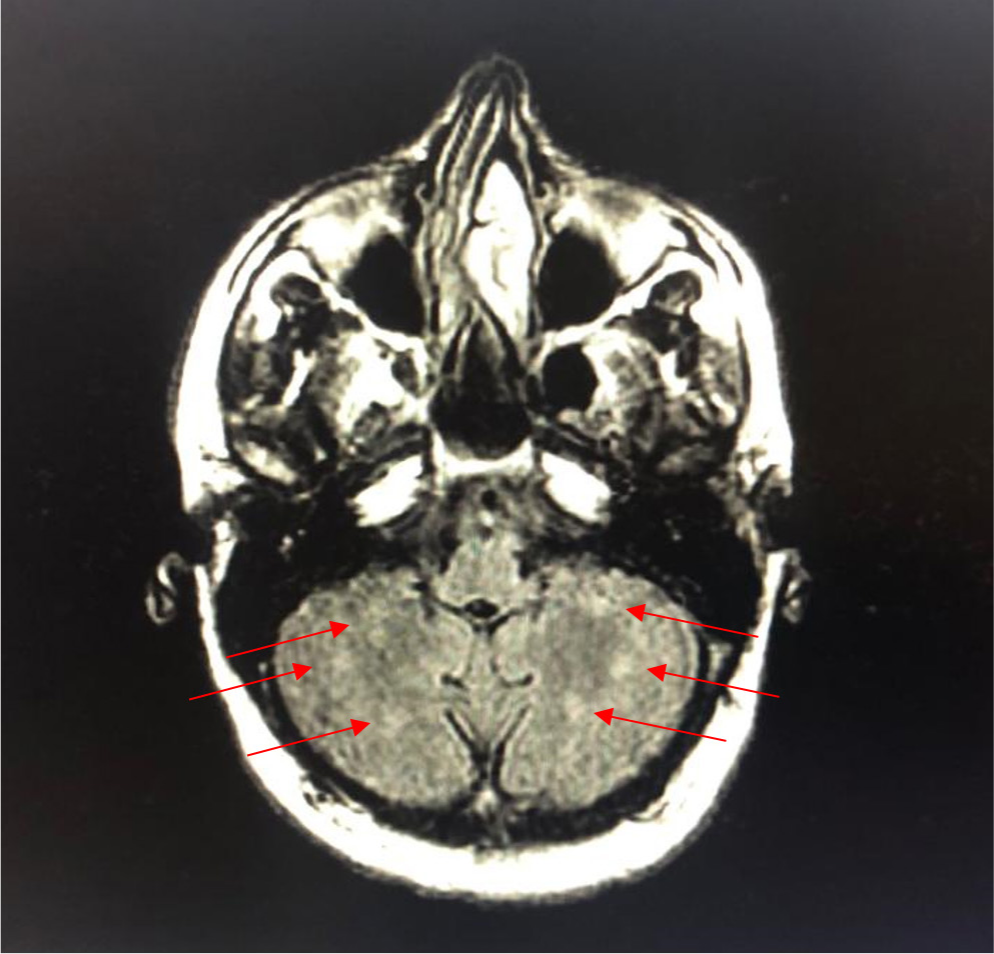
T2 FLAIR MRI of the brain in August year X showing multiple nodular signal abnormalities of the cerebellar hemispheres.

**FIGURE 2:**
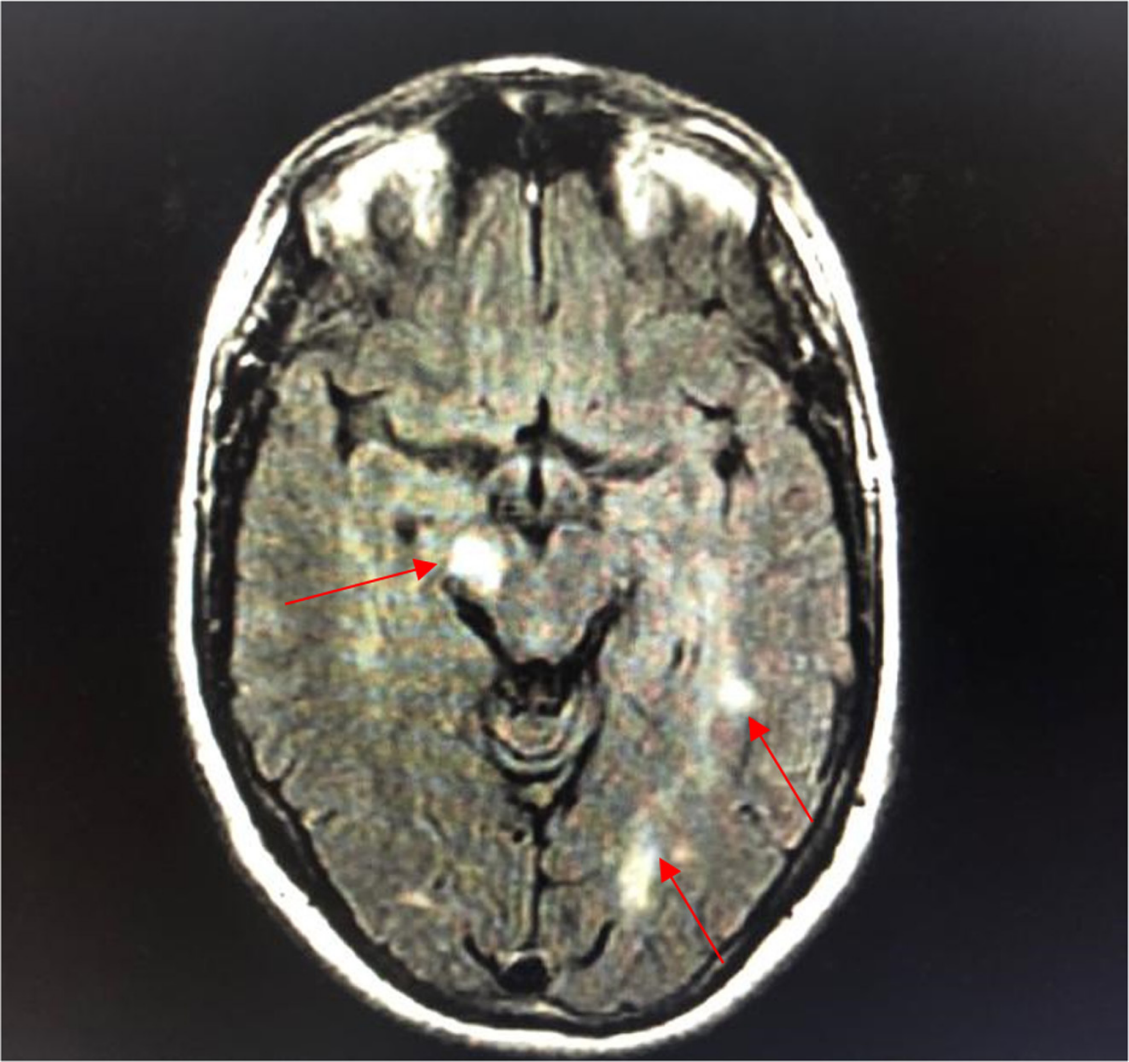
T2 FLAIR MRI of the brain in August year X showing signal abnormalities of the right midbrain and left temporal lobe.

**FIGURE 3:**
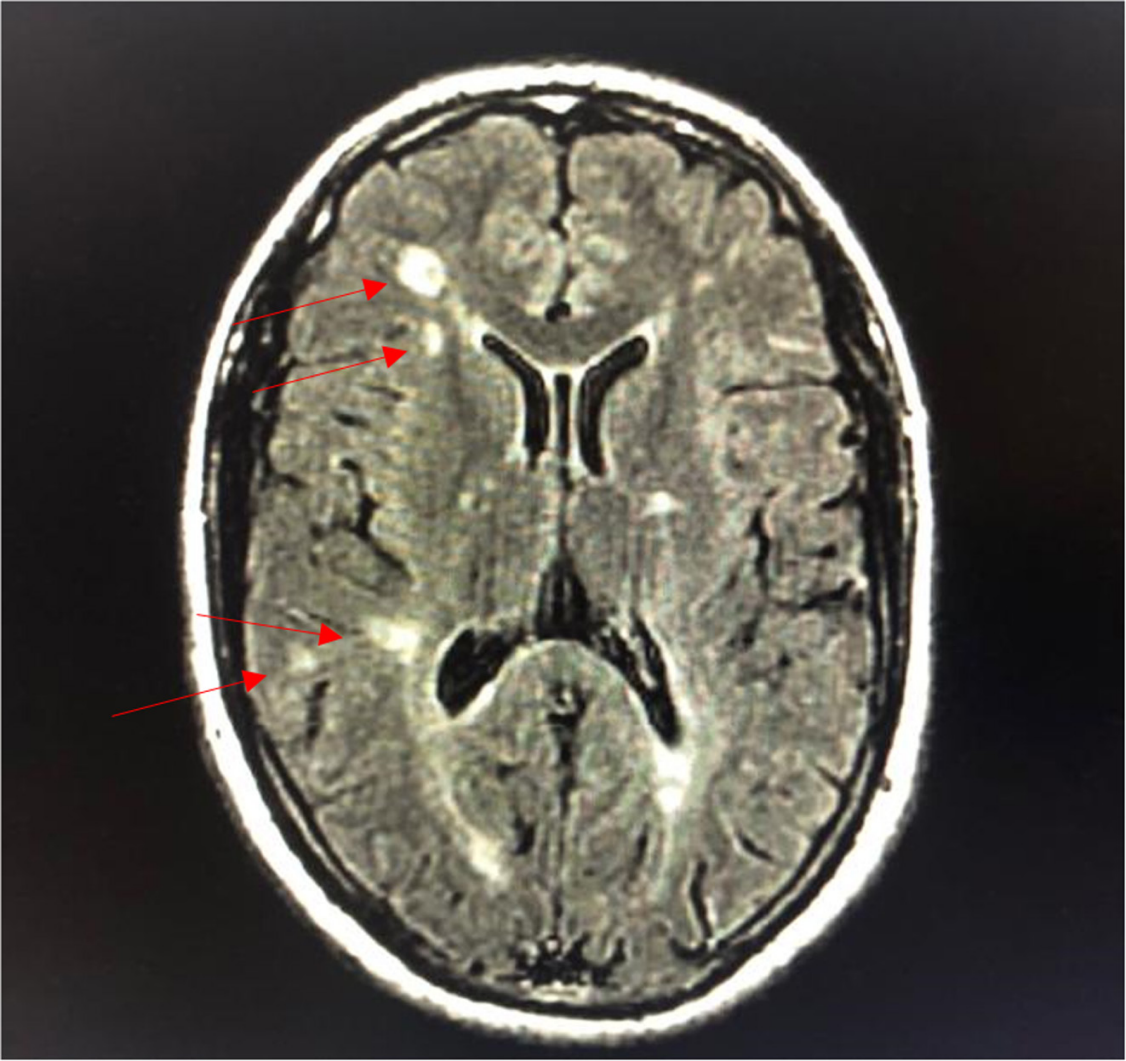
T2 FLAIR MRI of the brain in August year X showing signal abnormalities in the subcortical area of the frontal, parietal and temporal lobes, as well as the periventricular white matter.

Magnetic Resonance Imaging (MRI) of the brain done after neurology consultation revealed the presence of multiple nodular signal abnormalities of the cerebellar hemispheres (Figure 1), of the right midbrain and left temporal lobe (Figure 2), in the subcortical of the frontal, parietal and temporal lobes, as well as the periventricular white matter and the right cerebral peduncle (Figure 3) showing a T2 FLAIR hypersignal, T1 isosignal. A cervical MRI showed multiple segmental focal nodular signal abnormalities involving the lateral and posterolateral aspect of the medullary cord at the level of C3‐C4, facing C5‐C6 and at the cervico‐dorsal junction. These formations result in a T2 hypersignal and T1 isosignal, without enhancement on the injected sequences.

A lumbar puncture was carried during the hospital stay, showing the presence of oligoclonal bands (OCB), albumin in CSF of 43 mg/dL (10–30), IgG in CSF of 19, 5 mg/dL (<4,0), albumin in serum of 5000 mg/dL (3900–5000), IgG in serum of 1443 mg/dL (700–1500), and an elevated IgG index of 1.57 (0–0.85). The search for Anti‐MOG and Anti‐AQP4 antibodies was negative, which reinforced the diagnosis of MS.

A pulse therapy with Methylprednisolone 1000 mg IV once daily for 5 days resulted in significant improvement. Another episode of weakness after taking a hot shower prompted a new cerebral and cervical MRI 3 weeks after the initial one.

The brain MRI showed the presence of multiple signal abnormalities of the supratentorial and infratentorial subcortical white matter bilaterally as well as in the preventricular and the right cerebral peduncle, some of which were reduced in size compared to the previous examination. Some lesions presented enhancement and we noted the presence of signal abnormalities at the knee and isthmus of the corpus callosum.

**FIGURE 4:**
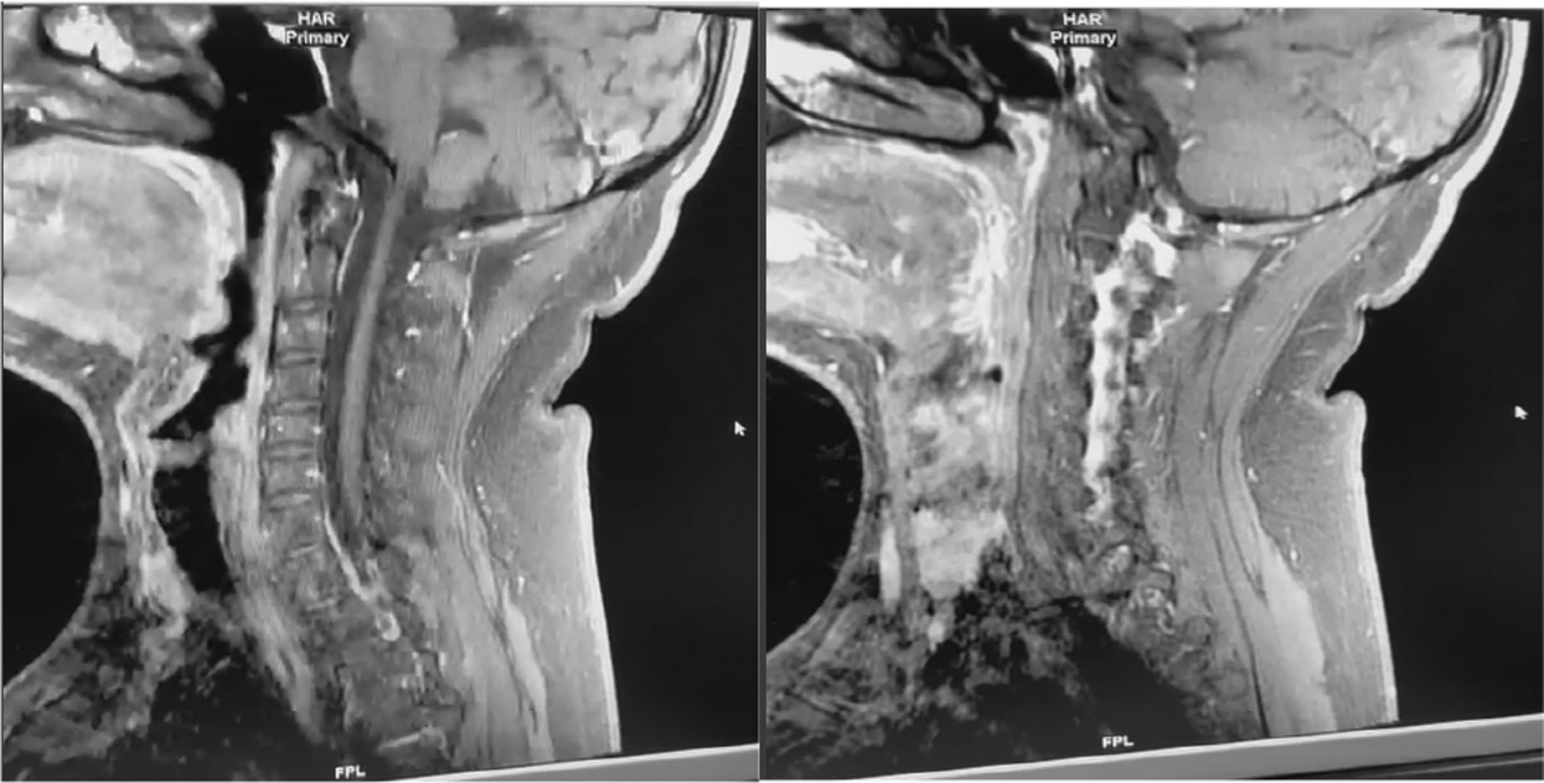
Cervical MRI with gadolinium injection, showing no enhancement of the lesions.

The cervical MRI showed multiple anomalies of segmental focal nodular signals of particular interest to the lateral and postero‐lateral levels of the spinal cord at the level of C2, notably C3 and C3‐C4, C6‐C7, and the cervico‐dorsal spinal junction. No enhancement on the sequences injected with Gadolinium. (Figure 4) These anomalies were still in favor of an inflammatory process in the white matter. The patient had frequent relapses and new MRIs were carried each time, confirming dissemination in time and space.

## 3 METHODS

The diagnosis of highly active relapsing remitting MS (RRMS) was thus confirmed as per the revised 2017 McDonald criteria,^5^ in coexistence with SLE. The patient was screened for other autoimmune diseases, and the panel was negative (IgG Anti‐Beta‐2GPB1, IgM Anti‐Beta‐2GPB1, IgG Anticardiolipin, IgM Anticardiolipin, IgG Anti dsDNA) and the ANA was slightly positive (1/160) as he was still taking hydroxychloroquine.

After the patient was screened for tuberculosis, hepatitis B and C and HIV, he was started on October year X, on subcutaneous interferon beta‐1a (Rebif) 44 microgram three times per week for 6 months. The patient did not respond to treatment, as he kept having frequent relapses (visual, motor, partial seizure) till February year X + 1 despite the injections, and was admitted many times for pulse therapy. The patient was shifted to second line therapy in April year X + 1: fingolimod (Gilenya) 0.5 mg orally once daily; the patient suffered of severe lymphopenia (grade 4) as a side effect.

## 4 CONCLUSION AND RESULTS

**FIGURE 5:**
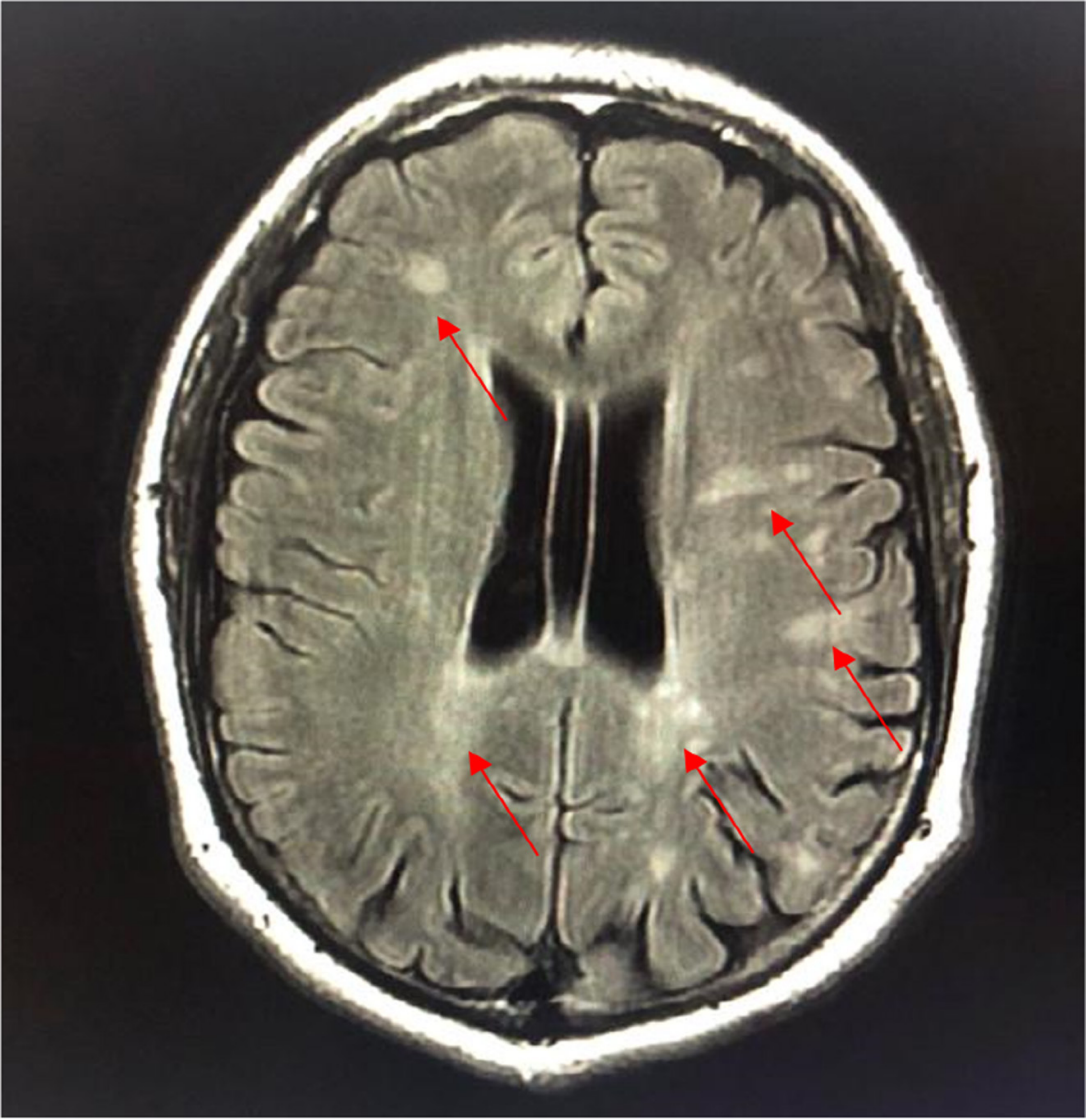
T2 FLAIR MRI of the brain in December year X+1 showing bilateral periventricular, right frontal oivoid subcortical and a lesion, which is perpendicular to the corpus callosum axis and parallel to the U fibers.

**FIGURE 6:**
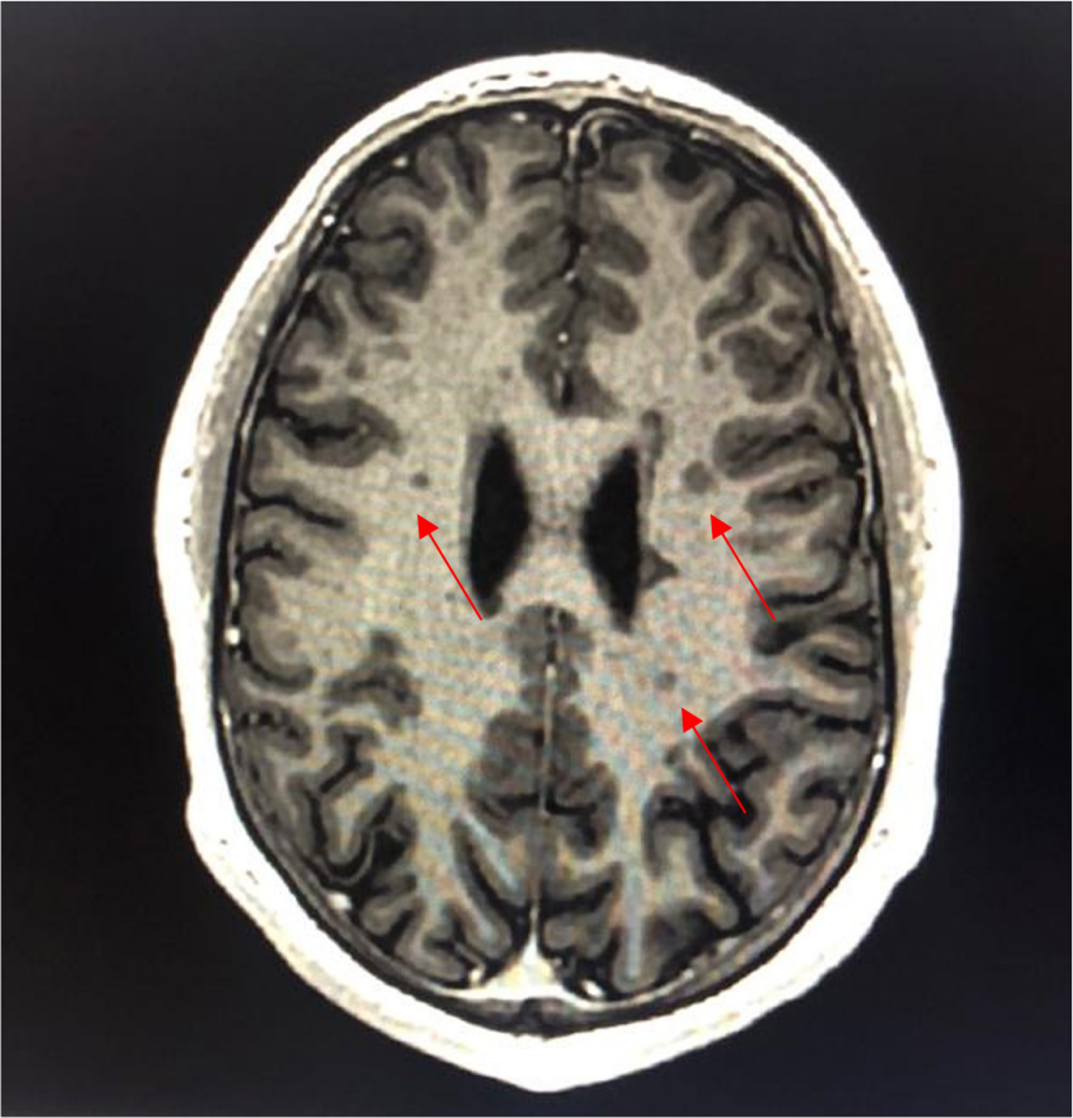
T1 MRI of the brain in December year X+1 showing black holes.

**FIGURE 7:**
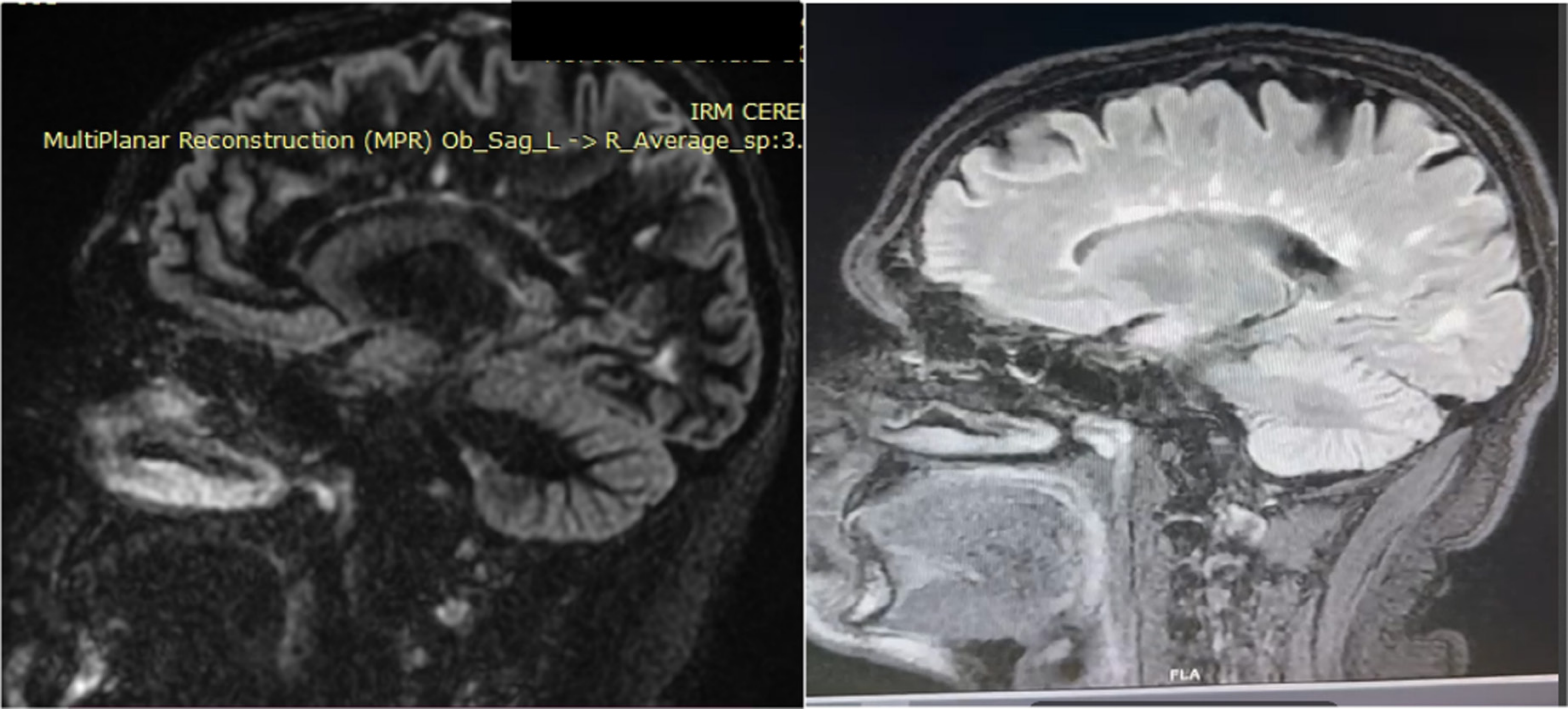
MRI year X+1 showing Dawson's finger sign.

**FIGURE 8:**
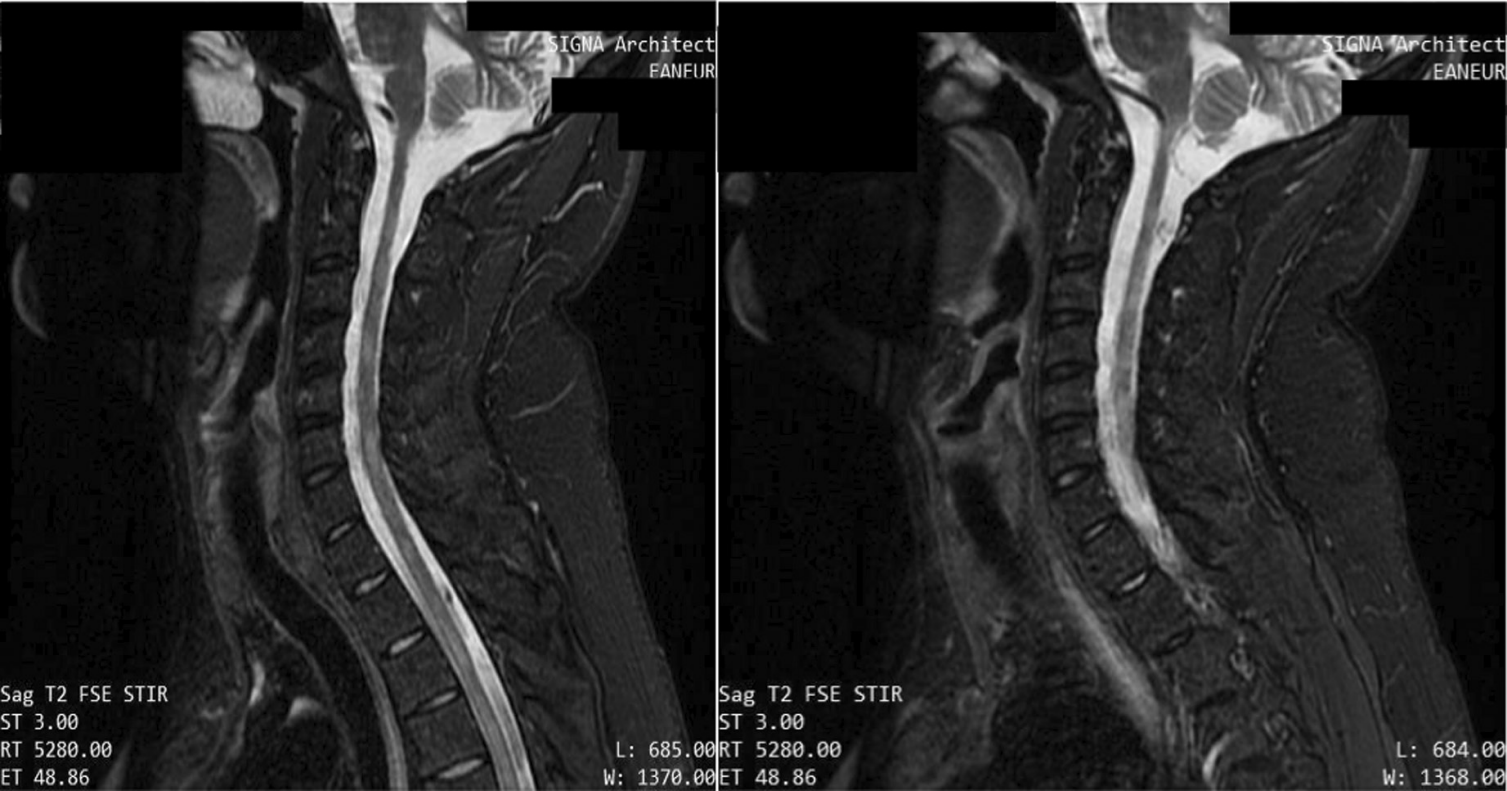
T2 STIR cervical MRI in April year X+2 showing hyperintense lesions.

MRI of the brain in December year X + 1 showed bilateral periventricular, right frontal ovoid subcortical lesion and a perpendicular lesion to the corpus callosum axis and parallel to the U fibers on T2 flair (Figure 5), and black holes on T1 (Figure 6) Escalation therapy was then prompted: Cladribine 10 mg orally. A total dose of 3.5 mg/kg was targeted. The patient weighed 80 kg which amounted to 80 kg × 3,5 mg = 280 mg, which equaled 28 tablets, which were given according to the following protocol: Two cycles of two courses each. The first cycle consisted of a course in February year X + 2, and a course in March year X + 2, of 5 days each. The patient took 2 tablets of the first and second day of each course, and 1 tablet on the third, fourth and fifth day. The patient experienced two relapses: one in March and one in November year X + 2. Dawson's fingers sign was clearly visible on an MRI conducted in December X + 1. (Figure 7) A subsequent MRI in April X + 2, showed new lesions consistent with MS on T2 flair along with hyperintense lesions on T2 STIR. (Figure 8).

The second cycle consisted of a 5‐day‐course in January year X + 3, during which the patient took 2 tablets on the first, second and third day, and one tablet on the fourth and fifth day, and a 5‐day‐course in February year X + 3. The expanded disability status scale (EDSS) was estimated to 0.5–1 by the end of the treatment, that is, outside the periods of acute exacerbation/relapse.

During acute exacerbations of the disease, the patient received methylprednisolone for 3 to 5 days, along with antiepileptic drugs. His symptoms included the following: ataxia/altered proprioception manifested by frequent dropping of objects, urinary incontinence, sexual dysfunction, optic neuritis, increased episodes of insomnia, hearing impairment and cognitive impairment (depression and memory loss, especially relatives' names).

The patient experienced a new relapse in October year X + 4. In December year X + 4, the patient's physician opted for Ocrelizumab IV 300 mg x 2 administered over 4 hours in a day hospital every 6 months. However, due to the unavailability of the drug, the patient was put on rituximab (Mabthera) 1000 mg IV every 6 months and hasn't experienced any exacerbations of the disease since, at the time of writing (March year X + 6). The patient still suffers from polyarticular pain and rash from the lupus. The renal function is conserved, he has a mild proteinuria (<150 mg/24 h).

## 5 DISCUSSION

### 5.1 SLE and gender

The pathogenesis of SLE has been strongly linked to sex hormones in animal models.^6^ In humans as well, exacerbations of SLE due to rapid hormonal changes are well documented (pregnancy, post partum, menses, etc.). There are also reports associating the use of estrogen‐containing oral contraceptives and disease exacerbations.^7^ However, it has been proven that men with lupus do not produce abnormal estrogen levels, which suggests that estrogens cannot be considered an isolated determinant of lupus.^8^ In a study of the androgenic status of 12 men with SLE, there was no evidence of hypogonadism or androgen deficiency. Men with lupus are thus fertile, sexually active and have normal reproductive history.^9^

### 5.2 MS and gender

On the other hand, sexual dysfunction is one of the most frequent disorders in MS patients. The role of hormones in the increased susceptibility for women of developing MS has been supported by the changes in disease course with alterations in sex hormones, especially in pregnancy, during which high levels of progesterone, oestradiol and oestriol are found,^10^, ^11^ which lead to an amelioration in the disease course of MS. Like MS, studies have proven that rheumatoid arthritis and psoriasis may improve during pregnancy,^12^, ^13^ whereas SLE may worsen.^14^, ^15^, ^16^, ^17^

In parallel, it has been reported that 24% of male MS patients tested had significantly lower levels of testosterone compared to age‐matched healthy men.^18^ This may endorse the hypothesis that endogenous androgens at physiologic levels are protective against autoimmune diseases, of which MS.^19^

Our patient was sexually active and started suffering from sexual dysfunction after the diagnosis of MS (which occurred 6 years later) and not of SLE, along with other symptoms (ataxia, incontinence, etc.). No hormonal tests (testosterone, estrogen levels, etc.) were carried in our patient.

### 5.3 Overlapping of MS and SLE

Our patient met the McDonald 2017 revised criteria for MS. He had more than two lesions in different regions, showing dissemination in space, and more than two relapses, proving dissemination in time. OCB were present in the CSF. There was no systemic involvement and serological tests for the exclusion of other diseases were negative, except for SLE. It is noteworthy that OCB can test positive in 15%–50% of patients with SLE. However, their presence in MS can be proven in 98% of cases.^20^, ^21^

Brain MRIs in patients with NPSLE could show small vessel disease/dementia (characterized by subcortical and periventricular white matter lesions on T2‐weighted sequences), cerebral atrophy, ischemia, hemorrhage or vasculitis. As for a demyelinating syndrome, although included in the initial American College of Rheumatology definition, it is now thought that presentations similar to MS, once termed ‘lupoid sclerosis’, are unlikely to be associated with SLE at all.^22^, ^23^, ^24^, ^25^

Jennekens et al. summarized the different forms of white matter damage in NPSLE (Table 1).^23^ On the other hand, in MS, the brain lesions on MRI are ovoid and periventricular and the corpus callosum is frequently affected; it is also common to see brain stem, subcortical and spinal cord lesions. In our case, some of the lesions had a periventricular distribution and were dynamic, which is more typical of MS: in contrast, the lesions in NPSLE are more static. So our patient's images exhibited features typical of MS.

**TABLE 1:** Different forms of white matter damage.^23^

Small punctate MRI lesions predominantly in the periventricular and subcortical white matter. Histology: small infarcts with loss of axons and myelin and with gliosis
2 Demyelinating plaques in brain and brainstem
3 Lesions of the optic nerves, and lesions of the spinal cord extending over two vertebrae or more, as in Devic's syndrome. Histology: vacuolar myelin degeneration, axonal loss, white matter necrosis
4 Extensive white matter lesions (leucoencephalopathy) as revealed by MRI or CT, in brain and brainstem, reversible in at least some cases, due to oedema or confluent small lesions

As far as we know, this is the initial documented instance of a male patient exhibiting concurrent SLE and MS. In the rare cases of MS and associated SLE in the same patient described so far, all the patients were female. Given the rare but possible association of MS and SLE, treatment that would address both diseases is desired. Rituximab seems to be a valid option for patients with MS and SLE as suggested in the literature review. Our patient has been free from relapses for 20 months, with 18 of those months being on rituximab treatment. Future studies investigating the gender distribution of patients with both MS and SLE, as well as exploring the underlying mechanisms and causes, would be valuable.

## AUTHOR CONTRIBUTIONS

**Tatiana Charbel:** Conceptualization; data curation; formal analysis; methodology; project administration; resources; supervision; writing – original draft; writing – review and editing. **Dany Akiki:** Conceptualization; data curation; formal analysis; methodology; project administration; resources; supervision; writing – original draft; writing – review and editing. **Said El Hage:** Conceptualization; data curation; formal analysis; methodology; resources; writing – original draft; writing – review and editing. **Gaby Moukarzel:** Conceptualization; data curation; formal analysis; investigation; methodology; project administration; resources; supervision; writing – original draft; writing – review and editing. **Elie Assaf:** Conceptualization; data curation; formal analysis; investigation; methodology; project administration; resources; supervision; writing – original draft; writing – review and editing.

## FUNDING INFORMATION

There is no funding to disclose for this case report.

## CONFLICT OF INTEREST STATEMENT

The authors have no conflicts of interest to disclose.

## ETHICS STATEMENT

Not required.

## CONSENT

Written informed consent was obtained from the patient to publish this report in accordance with the journal's patient consent policy.

## Data Availability

Data sharing is not applicable to this article as no new data were created or analyzed in this study.
